# Rationale, design and protocol of a longitudinal study assessing the effect of total knee arthroplasty on habitual physical activity and sedentary behavior in adults with osteoarthritis

**DOI:** 10.1186/s12891-016-1141-5

**Published:** 2016-07-13

**Authors:** Rebecca M. Meiring, Emmanuel Frimpong, Lipalo Mokete, Jurek Pietrzak, Dick Van Der Jagt, Mohammed Tikly, Joanne A. McVeigh

**Affiliations:** Exercise Physiology Laboratory, School of Physiology, Faculty of Health Sciences, University of the Witwatersrand, 7 York Rd, Parktown, Johannesburg, South Africa; Division of Orthopaedics, Faculty of Health Sciences, University of the Witwatersrand, 7 York Rd, Parktown, Johannesburg, South Africa; Division of Rheumatology, Department of Medicine, Chris Hani Baragwanath Academic Hospital, Faculty of Health Sciences, University of the Witwatersrand, 7 York Rd, Parktown, Johannesburg, South Africa; School of Physiotherapy and Exercise Science, Curtin University, Kent St, Bentley, Western Australia

**Keywords:** Accelerometery, Osteoarthritis, Sedentary behaviour, Physical activity, Knee arthroplasty

## Abstract

**Background:**

Physical activity levels are decreased and sedentary behaviour levels are increased in patients with knee osteoarthritis (OA). However, previous studies have shown that following total knee arthroplasty (TKA), objectively measured physical activity levels do not change compared to before the surgery. Very few studies have objectively assessed sedentary behaviour following TKA. This study aims to assess patterns of objective habitual physical activity and sedentary behaviour in patients with knee OA and to determine whether these change following TKA.

**Methods:**

Patients diagnosed with knee osteoarthritis and scheduled for unilateral primary total knee arthroplasty will be recruited from the Orthopaedic Division at the Charlotte Maxeke Johannesburg Academic Hospital. Eligible participants will have assessments completed one week before the scheduled arthroplasty, six weeks, and six months post-operatively. The primary outcomes are habitual physical activity and sedentary behaviour which will be measured using accelerometry (Actigraph GTX3+ and activPal monitors) at the specific time points. The secondary outcomes will be improvements in osteoarthritis-specific quality of life measures using the following questionnaires: Western Ontario and McMaster Universities Osteoarthritis Index (WOMAC), Knee injury and Osteoarthritis Outcome Score (KOOS), Oxford Knee Score (OKS), Knee Society Clinical Rating System (KSS), UCLA activity index; subjective pain scores, and self reported sleep quality.

**Discussion:**

The present study will contribute to the field of musculoskeletal health by providing a rich detailed description of the patterns of accumulation of physical activity and sedentary behaviour in patients with knee OA. These data will contribute to existing knowledge using an objective measurement for the assessment of functional ability after total knee arthroplasty. Although studies have used accelerometry to measure physical activity in knee OA patients, the data provided thus far have not delved into the detailed patterns of how and when physical activity is accumulated before and after TKA. Accurate assessment of physical activity is important for physical activity interventions that target special populations.

**Trial registration:**

NCT02675062 (4 February 2016).

## Background

Osteoarthritis (OA), the most common joint disorder, causing disability and loss of function, affects over 40 % of adults (aged 70 years and older) worldwide [1]. Concomitant with disrupted sleep, depression, increased sedentary behaviour, less physical activity, obesity, and polypharmacy, OA is associated with a decreased quality of life. Most occurrences of OA (41 %) are in the knee [2]. Although data are lacking from low to middle income countries, the few studies that have been done have shown that similar to populations from the US, knee OA is more common than is hip OA in African populations [3–5].

Pain is often a major contributing factor that impedes physical activity and reduces functionality in patients with knee OA [6, 7]. Approximately 80 % of individuals with OA experience limitations in movement and 25 % of individuals with OA experience limitations in major activities in their daily lives [7, 8] and the more severe the pain, the greater the degree of physical disability [9]. Conversely higher physical activity is associated with a lower risk of OA related joint pain and stiffness [10].

### People with knee OA are physically inactive and highly sedentary

Studies that have used objective measures to evaluate physical activity and sedentariness in patients with knee OA have found that 41.1 % of males and 56.5 % of females with knee OA are inactive [11], that is they do not meet the recommended physical activity guidelines [11–15]. In addition to the total amount of time spent in physical activity (PA) being important for health outcomes in adults [16, 17], there is an emerging body of evidence to show that the patterns of how physical activity and sedentary time are accumulated may also have implications for health. There remains a paucity of literature describing the detailed patterns of how physical activity is accumulated in patients with knee OA.

Sedentary behaviours (SBs) are those with an energy expenditure of less than 1.5 metabolic equivalents (METS) [18, 19]. In older men, interruptions to sedentary time is associated with better muscle quality in older men [20]. Adults with OA who are more sedentary experience a greater loss in functional capacity compared to adults who are less sedentary [12, 21–23], and this relationship appears to be independent of time spent in moderate to vigorous physical activity [21]. The number of daily hours patients with OA spend being sedentary (9.8 h) [12, 24] is similar to that reported in healthy adults (9.2 h) taking part in the European RISC study [25]. Additionally sedentary time in patients with knee OA is often elevated prior to knee replacement surgery [12, 26]. However detailed data describing how and when sedentary behaviour is accumulated are lacking in this population.

### Physical activity and total knee arthroplasty

Compared to the other rheumatic diseases, pharmacological treatment for OA is relatively unsatisfactory [27]. Although non-surgical treatment is the primary choice of treatment in OA patients [28], the main indication for total knee arthroplasty (TKA) is failure of conservative treatment; essentially pain that is not responsive to both pharmacological and non-pharmacological measures together with an increasing difficulty with activities of daily life in the context of advanced degenerative changes of the knee. There are no accurate figures for the number of knee replacements done in South Africa as there are no established registries. The main aim of the surgery is to alleviate pain and restore quality of life. Generally, patients are being operated on earlier in the progressions of the disease as it is recognized that quality of life is more likely to be restored in those patients, as opposed to patients with advanced disease where quality of life can be improved but not necessarily restored. As such regaining of functional ability allowing for restoration of habitual activity levels as near to normal as possible and a reduction in sedentary time are important goals for post-operative knee OA patients. Hence a desirable outcome that could be considered useful in assessing the regaining of functional ability would be to determine the number of patients who meet current physical activity guidelines [29] (including a reduction in sedentary behaviour) following TKA.

Physical activity and functional ability outcomes have historically been measured using self-report, but a more objective and quantified understanding of the impacts of knee arthroplasty on physical and functional ability are needed. Studies using interview based questionnaires have shown improved mobility benefits of TKA in developed countries [30], although data in low to middle income countries are scarce.

### Assessment of functional ability in knee OA patients following TKA

Studies using self-report have shown positive improvements in functional ability (ability to perform activities of daily living), pain and quality of life after TKA [31–33] while others have reported no significant improvements on health outcome measures following TKA [31, 34–36]. Self-report may also be open to bias and inaccuracy [37]. Thus there is a need for studies which use objective measures of physical activity as an important indicator of functional ability. Currently, habitual physical activity levels in large scale studies of healthy adult populations are most commonly objectively measured through the use of accelerometers [38].

Only nine studies since 2002 have used accelerometry as a measure of physical function in patients before and after TKA [39], some reporting little or no change in physical activity after surgery [24, 26, 33, 40] and others showing improvements in self-report measured functional ability [41, 42]. The variability in devices used to assess habitual physical activity before and after TKA makes the comparison of results across studies difficult. However, the ActiGraph GT1M accelerometer (an earlier version of the ActiGraph) has been used to assess the intensity and amount of physical activity occurring following TKA [26] but very small changes in activity were found. In addition, the timing and length of activity assessment is an important factor in determining changes in physical function in studies of TKA with some objectively measured studies showing improvements in physical activity six months after TKA [24, 43], and others showing very little or no improvement in daily activity at three or six months post-surgery [26, 41].

Recently, opportunities for measuring patterns of sitting and lying time have been made possible through the use of inclinometers e.g. the activPAL (PAL technologies Ltd, Glasgow, UK). The activPAL produces highly accurate and precise estimates of total sedentary time in free-living individuals [44] and has been validated to estimate time in different postures, step count, static and dynamic behaviours and sit-to-stand transitions in laboratory studies [44–46]. Only one study has used the activPAL in a cross-sectional assessment of energy expenditure in knee OA patients prior to arthroplasty [47]. The use of the activPAL to measure sedentary behaviour following TKA has not been done before.

The objective assessment of habitual physical activity may provide an alternative method of assessing whether functional ability is improved in OA patients following TKA, as a change in activities of daily living (assessed using sedentary behaviour and light activity measurements) without a change in moderate to vigorous physical activity may correspond to a patient’s improved ability to function. Thus the aims of this study are to 1) describe habitual physical activity and sedentary behaviour patterns in knee OA patients scheduled for TKA, 2) to investigate the effects of unilateral primary TKA on objectively and subjectively measured habitual physical activity, sedentary behaviour and health outcomes of patients with knee OA and 3) to determine whether subjective measures of functional ability and sedentary behaviour (questionnaires) are correlated with objective measures of habitual physical activity and sedentary behaviour (accelerometry) before and after TKA. These data will help inform targeted interventions for the improvement and maintenance of physical activity.

## Methods

### Study participants

The study population will include all knee osteoarthritis patients receiving care at the Charlotte Maxeke Johannesburg Academic Hospital. Patients will be recruited from the Orthopaedic Division of the hospital. The participants for this study will be knee OA patients scheduled (on surgical waiting list) for a single primary total knee arthroplasty or replacement surgery. Knee OA will be diagnosed based on clinical criteria as defined by the American Rheumatism Association (ACR) [48]. Prospective participants will be given an information sheet describing the study and will have the study verbally explained to them prior to participation in the study. Participants will be required to sign a consent form should they wish to participate in the study. Participants will then complete a general health questionnaire in order to confirm eligibility in the study. Patients will be recruited to participate in the study if they have been diagnosed with knee OA and attended to by surgeons in the Orthopaedic Surgery Unit at the Hospital. Potential participants will be eligible to participate in the study by the attending orthopaedic surgeon. They will also be included if they have been refractory to analgesics for at least six months, are male or female between 55 and 80 years of age, are undergoing primary unilateral TKA surgery and are ambulant with or without assistive devices.

Patients will be excluded from participating in the study if they use assistive ambulatory devices for mobility problems other than knee OA, are scheduled for bilateral knee arthroplasty, a second knee arthroplasty or revision, or are scheduled for total hip replacement, or if they have co-morbidities or medical conditions that affect physical activity such as congestive heart failure, stroke and other neurological problems, chronic obstructive pulmonary disease (COPD), gout and/or sepsis, have been diagnosed with arthritis other than osteoarthritis (according to the 1987 American College of Rheumatology (ACR) criteria [48]), if further joint surgery is anticipated within six months of the index knee replacement or are non-ambulant or wheel chair-bound.

### Study site

The study will be conducted at the Charlotte Maxeke Academic Hospital in Johannesburg, South Africa. It is an accredited central hospital with about 1088 beds serving patients from across Gauteng and neighbouring provinces. The hospital is situated in Parktown and also, serves as the main teaching hospital for the Faculty of Health Sciences, University of the Witwatersrand. Study participants will be recruited from the Division of Orthopaedics in the hospital. This hospital is chosen because: (1) it is a tertiary hospital that runs several specialist clinics including the Orthopaedic Division where TKA among several other surgeries are performed and (2) there is a collaboration between the Academic staff of the Faculty of Health Sciences of University of the Witwatersrand and the hospital Staff for teaching and research which will facilitate accessibility to patients.

### Study design

**Fig. 1:**
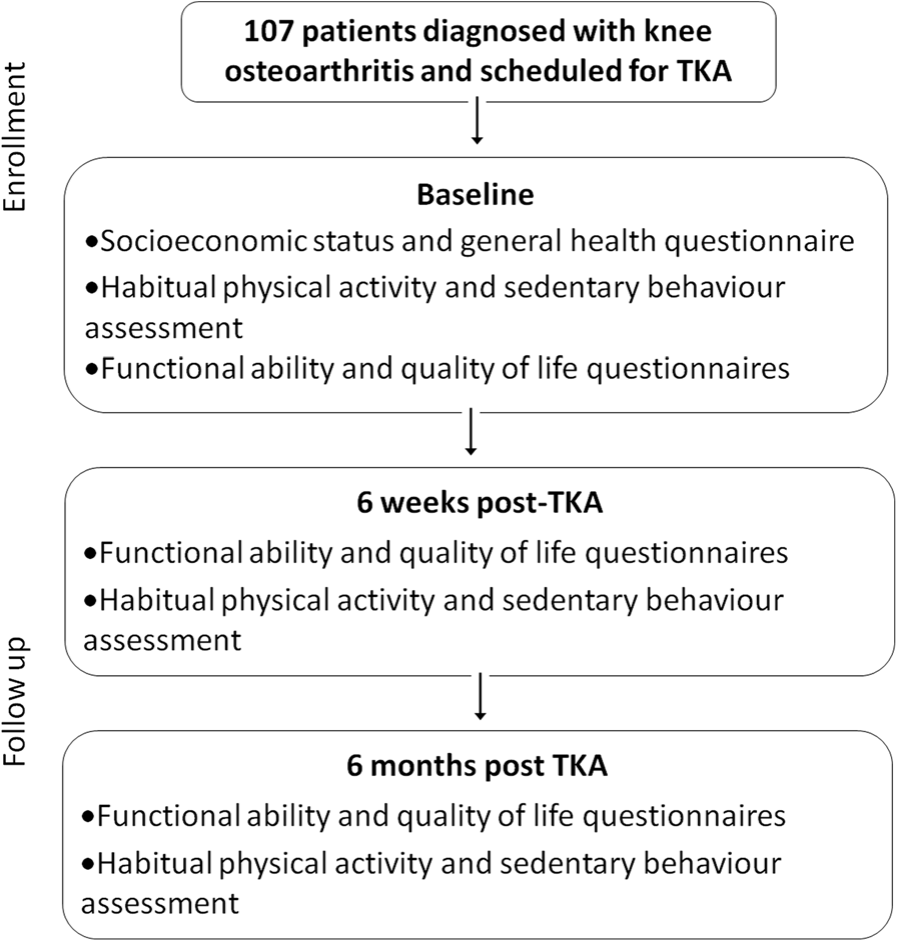
Flow diagram of time points for assessments of habitual physical activity, sedentary behaviour, functional ability, quality of life, general health, mobility and pain questionnaires before and after total knee arthroplasty (TKA)

This a longitudinal follow-up study of a cohort of participants who have been diagnosed with knee osteoarthritis and who are scheduled for TKA. After enrolment into the study, baseline assessments will be done prior to TKA. After TKA, participants will be followed-up and the same assessments done at baseline will also be done at six weeks, and six months post-operatively (Fig. 1). Habitual physical activity and sedentary behaviour will be measured using accelerometry (Actigraph GTX3+ and ActivPal monitors) at the specific time points. In addition, general health, functional ability, generic quality of life and pain questionnaires will be conducted at each time point on each participant.

### Trial registration

This trial has been registered with the ClinicalTrials.gov registry (trial registration number: NCT02675062).

### Outcomes

All outcomes will be measured at baseline, six weeks after surgery and again six months after the surgical intervention (Table 1). The primary outcomes of this study will be an improvement habitual physical activity and a reduction in sedentary time after TKA. The secondary outcomes will be improvements in osteoarthritis-specific quality of life measures: Western Ontario and McMaster Universities Osteoarthritis Index (WOMAC), knee related quality of life from Knee injury and Osteoarthritis Outcome Score (KOOS), Knee Society Clinical Rating System (KSS), the Oxford Knee Score (OKS), self reported activity using the UCLA activity index; subjective pain scores, and self reported sleep quality.

**Table 1:** Summary of outcome measures and respective collection method

Outcome	Variables	Measurement method	Time points (months)
Primary	Habitual physical activity	Accelerometry (ActiGraph worn on the hip)	0, 1.5, 6
	Sedentary behaviour	Accelerometry (activPAL worn on thigh)	0, 1.5, 6
Secondary	Knee OA-specific functional ability and quality of life	WOMAC	0, 1.5, 6
		KOOS	
		KSS	
		OKS	
	Activity	UCLA activity index	0, 1.5, 6
	Pain	VAS	0, 1.5, 6
	Sleep quality	Sleep questionnaire	0, 1.5, 6

### Sample size determination

Time spent in bouts of sedentary activity longer than 20 min is associated with poorer health outcomes [49]. In order to achieve a 2 % (which, for an average 16 h day, equates to a 20 min) reduction in the time spent in sedentary behaviour per day [26], a total sample of 107 participants will be required in this study to detect a significant effect of knee arthroplasty on sedentary behaviour (power of 80 %).

### Questionnaires

#### Socioeconomic Status (SES) and General Health Questionnaire (GHQ)

Socioeconomic status (SES) will be determined using a household amenity questionnaire [50]. Eligibility into the study will be determined using a GHQ in order to determine whether participants have any co-morbidities that might exclude them from participation. Furthermore the GHQ will be used to record the health demographics of the participants, such as history of knee OA and medication use. The SES and general health questionnaires will be completed at the first visit only.

#### Functional ability questionnaires

##### Western Ontario and McMaster Universities Osteoarthritis Index (WOMAC)

WOMAC assesses pain, stiffness, and physical function in patients with hip and knee OA (Bellamy, 2002). WOMAC consists of 24 items divided into 3 subscales: (1) 5 Pain items for assessing pain (2) 2 Stiffness items and (3) 17 Physical Function items. Each of the items requires the respondent to answer questions on 5-point Likert scale. These items request the respondent to indicate the degree of pain, stiffness, and physical functioning while engaging in specific activities, including walking, going upstairs, in bed at night, sitting, and standing upright. The scores for the items are summed and transformed to a 0-100 scale. Higher scores indicate improved pain, stiffness and physical function. Thus, a score of 0 represent the worst possible state while a score of 100 represents the best possible state.

##### Knee Injury and Osteoarthritis Outcome Score (KOOS)

The KOOS consists of 5 subscales: Pain, other symptoms, function in daily living, function in sport and recreation and knee-related quality of life. The previous week is the time period considered when answering the questions. Standardized answer options are given on five (5) Likert scales and each question is assigned a score from 0 to 4. The score is a percentage score from 0 to 100, with 0 representing extreme symptoms/problems and 100 representing no problems. The KOOS has been used in previous studies for evaluating outcome after TKA [51, 52].

##### Knee Society Clinical Rating System Score (KSS)

The Knee Society Clinical Rating System is a rating system which consists of two scores: knee and patient functional scores. Both scores range from 0 (worst health or functioning) to 100 (best health or functioning). This instrument has been used for tracking and reporting outcomes after total and partial knee arthroplasty worldwide [53]. Fifty of 100 points in the knee score are allocated to pain assessment with 50 representing no pain whereas the other 50 points are allocated for a clinical assessment with 50 representing at least 0°-125°of knee flexion with no active lag, no instability and normal alignment. The function score reflects patient-reported walking distance and stair-climbing and makes deductions for use of a walking aid, with 100 representing unlimited walking distance and normal stair-climbing without use of an aid [54].

##### Oxford knee score (OKS)

The OKS consists of 12 questions assessing pain and physical disability using a 5-point Likert scale and it generates a single score ranging from 0 (worst functional outcome) to 100 (best functional outcome) [55].

#### Physical activity questionnaire

The UCLA activity index is a scale from 1 to 10 with phrases (“no physical activity” to “regular participation in impact sports”) which the patient chooses to best describe their most appropriate activity level. The UCLA has been shown to be the most appropriate subjective physical activity assessment for patients undergoing joint arthroplasty [56].

#### Pain and sleep questionnaires

Joint pain, sleep quality and morning vigilance at each time point of the study will be assessed on a 100-mm visual analogue scale (VAS). The pain and sleep questionnaires will be administered at each visit to the hospital. All questionnaires have been previously validated.

### Anthropometric measurements

Height will be measured to the nearest millimetre with the participants bare foot using a stadiometer (Seca, model 202, Germany). Weight will be measured to the nearest gram using a scale (Mettler, Model TE120 ME36400, Switzerland) with the participants bare foot and wearing light clothing. Body mass index (BMI) will be calculated from the height and the weight (weight/square of height) for each participant.

### Physical activity and sedentary behaviour measurements

#### ActiGraph GT3X+ Accelerometer

The ActiGraph GT3X+ is a small (4.6 cm × 3.3 cm × 1.5 cm) lightweight (19 g) tri-axial activity monitor that provides data on physical activity including activity counts, energy expenditure (kcal), steps and activity intensity (METs). The GT3X+ has an inclinometer to determine body position in sitting, lying and standing. The ActiGraph GT3X+ will be worn by participants for 24 h/day for seven days at each of the assessment time points. It will be attached to an elastic nylon strap which the participants can wear as a belt around the waist on the side of the affected knee. Participants will be asked to remove the ActiGraph when showering, bathing or swimming. After seven days of accelerometer wear, the accelerometers will be collected at the next possible visit to the hospital or arrangement will be made for collection from participants at a location most convenient to them.

The data will be downloaded and processed using a custom built SAS program (v 9.3, SAS Institute, Cary, NC, USA) that implements a series of decision rules with user-modifiable thresholds to automatically identify waking wear data for continuously worn ActiGraph GT3X+ of 60 s epoch vertical axis data. Non-wear time will be classified as one minute intervals with consecutive zero counts for a minimum of 90 min (with an allowance of up to 3 min of counts between 0 and 50). For the day to be classified as valid, a minimum wear time of 10 h (600 min) is required. Each 60 s epoch of accelerometry data is classified according to calibration equations as sedentary if <100 counts per minute (cpm) [57], light intensity activity if between 100 and 1951 cpm, moderate-vigorous intensity activity if between 1952 and 5724 cpm and vigorous if >5724 cpm [58]. Adjacent epochs within the same intensity will be grouped into bouts. Only participants with four or more valid days of wear (including at least one weekend day) will be included in the analyses. Total daily time spent in the different PA intensities will be obtained by totalling the duration of all the bouts at each level for each day. The values will then be normalised to total wear time and averaged over the number of valid days to derive an estimate of the mean time spent within each intensity. A break in sedentary time will be counted as one minute or more where the counts per minute is greater than 100.

#### activPAL accelerometer

The activPAL is a small (2.0x1.4x0.3 in.) and light (20.1 g) single-unit accelerometer device worn on the mid-thigh fastened and secured by a non- allergenic adhesive tape and uses accelerometer-derived information about thigh position to estimate time spent in different body positions in lying, sitting and standing in 15 s epochs [44]. An activPAL will be taped to the thigh of the patient with waterproof taping and the patient will be asked to keep the activPAL on for the same amount of time as the ActiGraph. The activPAL can be covered with waterproof taping therefore, there will be no need to remove the activPAL when showering, bathing or swimming and therefore unless the device is reported to have fallen off by the patient, there will be no need for non-wear time classification for the activPAL data. The activPAL will be collected at the same time as the ActiGraph and data will be downloaded and analysed. The data are recorded in 15 s epochs and the manufacturer’s software will be used to determine the variables of interest from the downloaded activPAL data which will include the start time and duration of each sitting, lying, standing and stepping bout. Total sedentary time will be determined by summing the duration of all sitting/lying bouts. The interruption or break from sedentary time will be indicated at points where a sitting/lying bout is followed by a standing or stepping bout. Because the activPAL is worn for the same period as the ActiGraph, the same periods of sleep that are removed from the ActiGraph data, will also be removed from the activPAL data to be analysed. Further time-intensive methods of analysis will be employed according to current best practice guidelines [59].

### Data analyses

Socioeconomic data will be summarised by descriptive statistics. Also, means and standard deviations will be used to summarise data for age, anthropometric (height, weight, BMI and %body fat), ActiGraph GT3X+ and ActivPAL.Scores for WOMAC, KOOS, OKS and KSS will be calculated by their respective scoring systems. Data will be analysed using SPSS v20 (SPSS Inc., Chicago, IL, USA).

Linear mixed models will be used to determine relationships between objectively measured habitual physical activity and sedentary behaviour, functional capacity and quality of life between pre-operative and 6 weeks and 6 months post-operatively. The model will be used to compare subjective measures of functional mobility (questionnaires) with objective measure of habitual physical activity. A multiple regression analysis will be used to describe the association between physical activity and/or sedentary behaviour, functional capacity and quality of life of OA patients before and after TKA. Also, a multiple regression will be used to determine the relationship between habitual physical activity and sedentary behaviour on anthropometric parameters of OA patients before and after TKA. A *p*-value of less than 0.05 will be considered significant.

## Discussion

The present study will contribute to the field of musculoskeletal health by providing a rich detailed description of the patterns of accumulation of physical activity and sedentary behaviour in patients with knee OA. These data will contribute to existing knowledge using an objective measurement for the assessment of functional ability after total knee replacement surgery. Although studies have used accelerometry to measure physical activity in knee OA patients, the data provided thus far have not delved into the detailed patterns of how and when the entire spectrum of physical activity is accumulated before and after TKA. Accurate assessment of physical activity is important for physical activity interventions that target special populations.

A key advantage of this study over previous studies includes the use of more than one monitor to objectively assess physical activity and sedentary behaviour. The activPAL may be used to capture the energy expenditure of habitual physical activity, however the real value of the tool is its ability to assess sedentary behaviour in terms of postural changes (lying/sitting). Furthermore, the way we wish to obtain a richer set of metrics to describe patterns and accumulation of physical activity is rather more than the activPAL is able to provide, hence the reason why both monitors will be used in this study. Detailed postural classifications of how patients with knee OA spend their time prior to and after surgery will be of interest to health care providers. An increase in sedentary behaviour leads to an increased risk of sarcopenia in healthy elderly populations [60]. The muscle degeneration that is associated with sedentary behaviour in an ageing population has implications for patients with knee OA, as the immobility and reduced functionality may be exacerbated by the knee OA. Furthermore greater levels of sedentary behaviour may occur at an earlier age in patients with knee OA predisposing these patients to an increased risk of sarcopenia as they age. No studies that we are aware of have investigated sedentary behaviour patterns (using the activPAL) in patients with knee OA therefore comprehensive measurement on patterns of sedentary behaviour in this population is needed to implement effective interventions aimed at decreasing sedentary behaviour levels.

Additionally, there are no data on objectively measured habitual physical activity or sedentary behaviour before and after total knee replacement on South African patients. Surgical intervention may be considered at an earlier stage of the disease in developed countries, compared to low to middle income countries such as South Africa where surgical intervention may often be delayed due to long waiting lists and budgetary constraints. Knowing more detailed information about how patterns of physical activity and sedentary time may change following surgical intervention will help inform best practices by providing clear information about whether patients can expect to improve their physical activity levels and decrease their time spent in sedentary behaviours by enough time so as to reduce their risk of cardiometabolic disease (which is a risk associated with a lack of activity). Describing changes in daily patterns of activity and sedentary behaviour may allow for further studies to target the timing and duration of physical activity interventions on a daily basis in order to attempt to increase the amount of physical activity patients with knee OA take part in.

As global populations age and in order to decrease the burden of the disease, there will be an increasing need for total knee replacements in adults affected by knee OA. This study will investigate the patterns by which habitual physical activity and sedentary behaviour are accumulated in patients with knee OA before and after surgery, and will therefore assist in informing targeting interventions to improve patterns of physical activity. Furthermore, a change in exercise guidelines from increasing the volume of exercise to offsetting the time spent in deleterious behaviours such as excessive time spent sitting, may contribute to developing more relevant criteria for physical activity prescription in patients with osteoarthritis.

## Abbreviations

KOOS, Knee Injury and Osteoarthritis Outcome Score; KSS, Knee Society Clinical Rating System; METS, metabolic equivalents; OA, osteoarthritis; OKS, Oxford Knee Score; PA, physical activity; SB, sedentary behaviour; SES, socioeconomic status; TKA, total knee arthroplasty; WOMAC, Western Ontario and McMaster Universities Osteoarthritis Index
